# The PPARα and PPARγ Epigenetic Landscape in Cancer and Immune and Metabolic Disorders

**DOI:** 10.3390/ijms221910573

**Published:** 2021-09-30

**Authors:** Jesús Porcuna, Jorge Mínguez-Martínez, Mercedes Ricote

**Affiliations:** Myocardial Pathophisiology Area, Centro Nacional de Investigaciones Cardiovasculares (CNIC), 28029 Madrid, Spain; jesus.porcuna@cnic.es (J.P.); jorge.minguez@cnic.es (J.M.-M.)

**Keywords:** PPARs, cancer, immunity, obesity, diabetes, miRNA, DNA methylation, histone modification

## Abstract

Peroxisome proliferator-activated receptors (PPARs) are ligand-modulated nuclear receptors that play pivotal roles in nutrient sensing, metabolism, and lipid-related processes. Correct control of their target genes requires tight regulation of the expression of different PPAR isoforms in each tissue, and the dysregulation of PPAR-dependent transcriptional programs is linked to disorders, such as metabolic and immune diseases or cancer. Several PPAR regulators and PPAR-regulated factors are epigenetic effectors, including non-coding RNAs, epigenetic enzymes, histone modifiers, and DNA methyltransferases. In this review, we examine advances in PPARα and PPARγ-related epigenetic regulation in metabolic disorders, including obesity and diabetes, immune disorders, such as sclerosis and lupus, and a variety of cancers, providing new insights into the possible therapeutic exploitation of PPAR epigenetic modulation.

## 1. Introduction

### 1.1. Peroxisome Proliferator Activated Receptors

Peroxisome proliferator-activated receptors (PPARs) are a group of nuclear receptors (NRs) that act as ligand-activated transcription factors (TFs) [1]. Upon ligand binding, PPARs assemble with retinoid-X-receptors (RXRs), generating dimeric complexes that bind response elements in target genes to exert important regulatory functions [2]. PPARs are well known for their important functions in lipid and glucose homeostasis, nutrient sensing, inflammation, cellular differentiation, and development [3]. There are three PPAR isoforms: PPARα (NR1C1), PPARβ/δ (NR1C2), and PPARγ (NR1C3). The three PPAR isoforms are differentially expressed in distinct tissues and, more importantly, play different and contrasting roles upon ligand activation [4,5]. PPARα is expressed in tissues with high rates of fatty-acid catabolism, such as the liver, where it is mainly expressed. PPARα decreases glycolysis and lipogenesis, while enhancing glucose uptake, glycogen synthesis, and fatty acid oxidation. Although the PPARβ/δ isoform is expressed ubiquitously, its expression is prominent in the gastrointestinal tract and muscle, where it controls metabolism, glucose utilization, and lipid transport. PPARγ is mostly expressed in adipose tissue, where it promotes lipogenesis and adipocyte differentiation. It also improves insulin secretion by pancreatic β-cells, skeletal muscle sensitization to insulin, and gluconeogenesis in the liver.

Like other NRs, PPARs have a well-conserved structure. Between the N-terminal and C-terminal ends are a DNA binding domain (DBD), a flexible hinge, and a ligand-binding domain (LBD) [2]. The DBD includes a structure containing two zinc-fingers that recognize specific DNA sequences in the peroxisome proliferator response elements. These sequences consist of direct repeats of the hexanucleotide AGGTCA separated by a single nucleotide spacer [6]. The LBD contains 13 alpha helices and one four-stranded beta sheet and can interact with several ligands that activate or repress PPAR action [5,7]. Many natural and synthetic lipophilic acids are PPAR ligands, prominent among which are a wide variety of unsaturated fatty acids (docosahexaenoic and eicosapentanoic acids) and eicosanoids. Natural ligands include leukotriene B4 for PPARα and prostaglandin PGJ2 for PPARγ [5]. PPARα is also stimulated by a family of chemicals known as fibrates, such as fenofibrate and clofibrate. Similarly, PPARγ binds a group of synthetic molecules called thiazolidinediones (TZDs), including rosiglitazone and pioglitazone.

**Figure 1:**
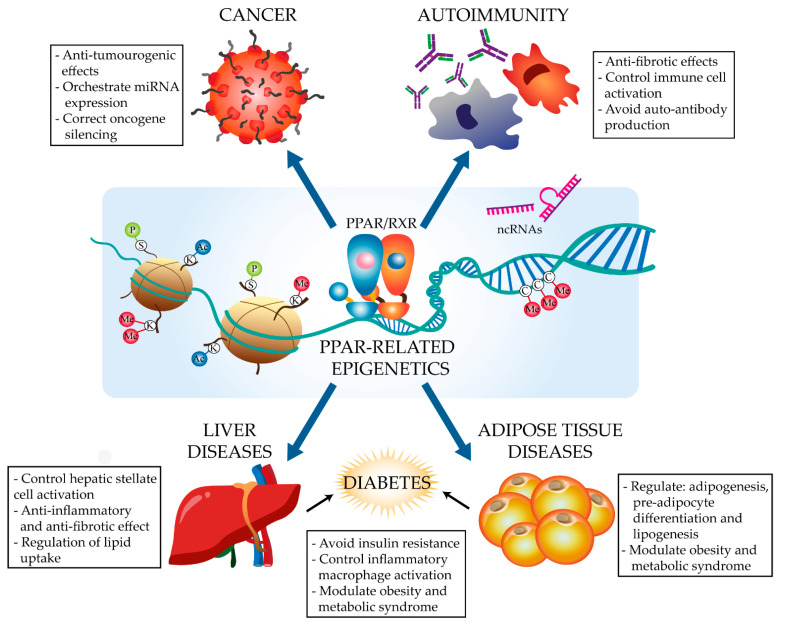
Scheme: PPAR epigenome implications in diseases. C, cytosine; Me, methyl group, ncRNAs, non-coding RNAs; K, histone lysine; S, histone serine; Ac, acetylation mark; P, phosphorylation mark.

PPARs regulate energy metabolism and inflammation, exerting anti-fibrotic and anti-inflammatory effects in diverse conditions, including cancer, autoimmune diseases, liver steatosis, and type 2 diabetes (T2D) [8,9,10]. PPARs stimulate the expression of anti-inflammatory molecules and inhibit the production of extracellular matrix proteins and pro-inflammatory cytokines, as well as modulating the response and phenotype of immune cells such as macrophages and lymphocytes [10]. The activation of all three isoforms has been demonstrated to polarize macrophages to an anti-inflammatory M2 phenotype and to regulate CD4+ T cell survival and differentiation towards different Th and Treg lineages [11,12]. PPARγ has been demonstrated to act as a key transcription factor in alveolar macrophage and osteoclast identity and ontogeny [13]. While the PPAR pathways implicated in the control of these processes are well characterized [10], little is known about the epigenetic modulation of or by PPARα and PPARγ. Nevertheless, recent research has begun to identify features of the PPAR epigenome in different diseases (Figure 1). These advances, together with the amenability of PPARs to ligand-modulation and the increasing availability of synthetic ligands [5], are driving the study of the complex transcriptional and epigenetic regulation of PPARs in specific diseases. Here, we discuss recent advances focused on the PPARα- and PPARγ-related regulation of non-coding RNAs, histone modification, and DNA methylation in the context of cancer and metabolic and immune-related disorders, as well as the emerging therapeutic potential of these processes in these diseases.

### 1.2. Epigenetics

The term epigenetics was coined by Conrad Hal Waddington in 1942 to explain the link between genes and the environment. Epigenetics is the study of mechanisms of stable and heritable gene regulation that require no changes to DNA sequence and can be defined as the set of environmental influences that determine a phenotype [14]. The three main epigenetic mechanisms are DNA methylation, histone modification, and the binding of non-coding RNAs to regulatory elements. These mechanisms perpetually modulate gene expression states in order to ensure the correct cellular fate and state without altering the DNA sequence.

#### 1.2.1. Major Epigenetic Modifications

##### DNA Methylation

DNA methylation, one of the most studied epigenetic modifications, is the addition of a methyl group (-CH3) to the fifth carbon atom of the cytosine ring, generating 5-methylcytosine (5meC). DNA methylation inhibits gene transcription [15] and is catalysed by the m5C DNA methyltransferase (DNMT) family of enzymes. These are classified into three groups, DNMT1, DNMT3a, and DNMT3b, which together establish and sustain the correct DNA methylation patterns. DNMTs, together with partners such as UHRF1 (ubiquitin like with PHD and ring finger domains 1), must be tightly regulated to avoid pathological outcomes, for instance the expression of oncogenes [16,17]. DNA methylation is a reversible epigenetic mark, and the removal of methyl groups is catalysed by the ten-eleven translocation (TET) methylcytosine dioxygenases [18].

##### Histone Modification

Histones are composed of the protein subunits H2A, H3, H3B, and H4 and act as cores around which DNA winds to form nucleosomes, the building blocks of chromatin. Histones can be modified by acetylation, methylation, ubiquitination, or phosphorylation. These specific modifications, or a combination of them, change the nucleosome conformation, thereby regulating access by the transcriptional machinery to the coding DNA [19,20,21]. The most well-studied histone modifications are acetylation and methylation [22]. Histone acetylation is the most frequent histone modification and is regulated by histone acetyltransferases (HATs) and histone deacetylases (HDACs). When histones are acetylated, the chromatin adopts a more relaxed and open conformation, allowing access to the gene transcription machinery. Histones can be methylated on lysines and arginines by histone methyltransferases (HMTs), with the reverse reaction catalysed by histone demethylases (HDMs) [23,24]. Histone methylation most often induces gene silencing by promoting the recruitment of DNMTs, followed by methyl-binding proteins and finally HDACs [25,26]. Nevertheless, histone methylation can promote the activity of positive transcriptional regulatory elements, such as de novo and pre-disposed enhancers or promoters [27,28,29].

##### Non-Coding RNAs

Non-coding RNAs (ncRNAs) are RNA molecules that do not translate into proteins but instead play important roles in gene expression regulation both transcriptionally, at the DNA level, and post-transcriptionally, at the mRNA level [30]. ncRNAs are a diverse group of molecules, and it is difficult to make general statements about their function and regulation [31]. The most widely studied ncRNAs in relation to epigenetics are micro RNAs (miRNAs) and long ncRNAs (lncRNAs). miRNAs are typically 18–24 nucleotides long and bind to complementary sequences in target mRNAs, marking them for degradation and thus preventing their translation into protein. lncRNAs, which can exceed 200 nucleotides, have a diverse interactome that includes DNA, proteins, peptides, mRNAs, and miRNAs, through which they regulate both transcription and translation [32].

The epigenome—the complete set of epigenetic marks—must be tightly regulated not only to sustain development and cell fate, but also to prevent pathogenic conditions that could otherwise arise at any moment during life [33]. The dynamic gene regulation afforded by epigenetics ensures a locally appropriate accessibility of chromatin to TFs and, therefore, the execution of a precise transcriptional program [34]. Given the importance of epigenetics for sustained gene transcription, all epigenetics programs within a cell need to work correctly in order to maintain cell function and phenotype, and to prevent possible inflammatory conditions derived from an altered epigenetic landscape [35]. Much recent research interest therefore focuses on the roles of specific epigenetic proteins, such as enzymes and TFs, in a range of biological processes, such as immune metabolism, inflammation, disease, and differentiation. Aberrant DNA and histone methylation, abnormal histone acetylation patterns, and altered ncRNA regulation have been linked to conditions, such as aging, neurological and metabolic disorders, allergies and other autoimmune diseases, and cancer [36,37,38,39,40,41,42,43,44,45,46,47]. Finding molecules able to modulate the epigenome would open up opportunities to specifically treat these conditions.

## 2. The PPARα and PPARγ Epigenetic Landscape in Disease

### 2.1. Cancer

PPARs have well known anti-tumourogenic effects [8]. PPARα activation can induce apoptosis and tumour cell death, preventing tumour expansion and inflammation. PPAR-related effects on tumour development have historically been linked to cell-cycle blockade genes such as p18, p21, and p27, leading to apoptosis through the inhibition of B-cell lymphoma 2 (Bcl-2) and reduced angiogenesis through the inhibition of vascular endothelial growth factor (VEGF) [48,49]. The implication of PPAR-related miRNAs and DNA modifications in tumour development has spurred interest in their potential as biomarkers and therapeutic targets. However, the evidence is disputed for some cancers and PPAR isoforms (Table 1).

#### 2.1.1. Colorectal Cancer

Colorectal cancer (CRC) accounts for 7–10% of incident cancers and 3.2% of all cancer-related deaths worldwide, and the incidence is increasing in developed countries [50]. Several studies have shown that PPARγ plays a protective role in CRC and have described the pathways involved downstream of PPAR, opening up the possibility of using PPAR agonists to treat CRC [51]. However, less is known about the upstream pathways and epigenetic mechanisms involved in the action of PPARs in CRC, and research in this area is ongoing.

CRC is often associated with obesity, and the tissue hypoxia characteristic of obesity has been linked to altered expression of typical CRC miRNAs [52]. In 2017, Motawi et al. reported that PPARγ epigenetic regulation contributes to the CRC risk of obese patients [53], showing that obese CRC patients have upregulated expression of the miRNAs miR-27b, miR-130b, and miR-138. In line with the anti-tumourogenic role of PPARγ in CRC, the expression level of these miRNAs correlated negatively with PPARγ mRNA and protein expression, possibly as a result of direct targeting of PPARγ mRNA [53]. Motawi and coworker’s findings are strongly supported by several previous studies [54,55,56,57,58]. However, others reported downregulated expression of miR-27b and miR-138 in colonic cancer cells and tissues [59,60,61], although none of these studies discussed PPARγ. Interestingly, miR-506, which is frequently dysregulated in cancer, has been shown to inhibit PPARα expression in the hydroxicamptothecin-resistant colon cancer cell line SW1116 [62]. Moreover, targeted downregulation of PPAR signalling pathway by a set of miRNAs has been reported in CRC-derived liver metastasis [63]. Together, these findings suggest the therapeutic potential of targeting PPAR-interacting miRNAs in CRC.

There is also evidence for a role in CRC of PPAR-related DNA methylation. UHRF1 was demonstrated to foster *Pparg* promoter methylation and repressive histone modifications that suppress PPARγ expression in human-derived CRC cell lines [64]. These in vitro results are in step with studies in CRC patients reporting an association between increased methylation of Pparg [65] and PPARγ target genes [66] and decreased PPARγ expression [67]. Furthermore, hypermethylation of the Pparg promoter suppressed PPARγ expression and was associated with CRC regardless of patient body weight [53]. Interestingly, PPARα acts as a suppressor of colon carcinogenesis in mice and is downregulated in mouse colonic tumours. Mice lacking PPARα had increased expression of DNMT1 and protein arginine methyltransferase 6 (PRMT6), resulting in methylation of the tumour suppressor genes P21 and p27, respectively [68]. However, recent evidence indicates that PPARα, along with PPARδ, is overexpressed in human CRC [69]. The inconsistency between these studies could be explained by the significant differences in PPARα expression and function between mice and humans [70].

#### 2.1.2. Liver Cancer

The most common type of primary liver cancer in humans is hepatocellular carcinoma (HCC), which is the third deadliest cancer in the world. A recent analysis of mouse and human single and bulk RNA-seq data revealed that PPARγ controls the expression of a set of antifibrotic miRNAs, including miR-30, miR-29c, and miR-338, that are important for the maintenance of low profibrotic protein levels during HCC-related liver fibrosis [71]. Conversely, other studies have reported that miRNA regulation of the PPAR pathway may contribute to HCC progression. For example, miR-27a inhibits the expression of PPARγ in hepatocarcinoma cells [72]. Interestingly, miR-27a also inhibits RXRα, possibly contributing to cell proliferation in rhabdomyosarcoma [73]. Given that PPAR forms obligate heterodimers with RXRs to regulate transcription, RXR-targeting miRNAs, like miR-27a and miR-34a [74], might also modify the binding capacity and activity of PPAR indirectly. One of the most differentially expressed miRNAs in human HCC samples is miR-9 [72,75], which has been shown to favour tumour growth and aggressiveness. Moreover, bioinformatic analysis identified putative miR-9 binding sites in the PPARα 3′UTR. However, it remains uncertain whether miR-9 contributes to the regulation of PPARα expression in HCC [75]. 

#### 2.1.3. Other Cancers

PPARγ has been proposed as a therapeutic target in thyroid cancer [76], but although attempts have been made to correlate PPARγ expression with miR-27a, as yet there is no firm evidence linking miRNAs and PPARs in this type of cancer [77]. PPARs are also plausible therapeutic targets in lung cancer. In canine primary lung cancer cells, the Pparg promoter shows a significant loss of 5′-methylation. However, although PPARγ is highly expressed in canine non-small lung cancer cells, this change in the methylation pattern was unrelated to the observed changes in PPARγ protein expression [78]. PPARγ is also dysregulated in gingivo-buccal oral squamous cell carcinoma (OSCC-GB), with OSCC-GB patients showing significant differential methylation of the PPAR pathway genes Cd36, Cyp27a1, Olr1, and Pparg itself. The anti-cancer potential of targeting PPARs is highlighted by the finding that synthetic PPARγ ligands can reduce the incidence of carcinogen-induced tongue tumours [79]. However, current PPARγ ligands are cytotoxic. As an alternative, interest has emerged in the epigenetic action of DNA methyltransferase inhibitors (DNMTI), which is able to renew the transcription of key silenced genes in this cancer, including Pparg [79]. However, as yet, there have been no reports on the molecular mechanism underlying DNA methylation and PPARγ regulation in these tumours. In 1.25-dihydroxyvitamin D3-treated human prostate adenocarcinoma cells, expression of miR-17/92 correlated with PPARα downregulation. However, a direct effect of miR-17/92 on PPARα expression has not been demonstrated experimentally [80]. 

**Table 1:** PPAR epigenetics in different cancers.

Condition	PPAR Isoform	Epigenetic Player	Effect	References
Colorectal cancer	PPARα	miR-506	PPARα expression inhibition in a hydroxicamptothecin resistant colon cancer cell line.	[62]
		DNMT1	Absence of PPARα caused P21 and P27 methylation by DNMT1.	[68]
	PPARγ	miR-27b, miR-130b and miR-138	Potential downregulation of PPARγ.	[53]
		UHRF1	Epigenetic PPARγ inactivation in human-derived CRC cell lines.	[64]
		Promoter hypermethylation	Hypermethylation of *Pparg* promoter caused PPARγ suppression.	[53]
Hepatocellular carcinoma	PPARα	miR-9	Putative biding sites to PPARα 3’ UTR.	[75]
	PPARγ	miR-30, miR-29c and miR-338	Antifibrotic miRNAs regulated by PPARγ during HCC-related liver fibrosis.	[71]
		miR-27a	PPARγ inhibition in hepatocarcinoma cells.	[72]
Thyroid cancer	PPARγ	miR-27a	no relation obsrved yet.	[77]
Lung cancer	PPARγ	Promoter methylation	Significantly loss of 5′-methylation.	[78]
Gingivo-buccal oral squamous cell carcinoma	PPARγ	DNMTs	DNA methyltransferase inhibitors could renew PPARγ transcription.	[79]
Prostate cancer	PPARα	miR-17/92	Possible direct PPARα targetting and dowregulation.	[80]

Thus, although research is uncovering new PPAR epigenetics-related factors with potential for the treatment of different types of cancer, much of the evidence has been obtained in vitro or consists of observational data obtained from patient samples. Much further research is therefore needed before the field can contemplate moving to cancer clinical trials of therapies based on the modulation of PPAR epigenetics.

### 2.2. Immune Disorders

PPARs, especially PPARγ, contribute to the suppression of key pro-inflammatory genes such as NF-kB, INFγ, TNFα, TGFβ, and the interleukins IL-1a and IL-6 [1,10]. These actions are related to the key roles played by PPARs in autoimmune diseases, such as celiac disease [81] and lupus [82]. In sepsis patients and in LPS-treated THP-1 cells PPARγ has been shown to upregulate miR-142-3p. This miRNA targets the 3′-UTR of high mobility group box-1 (HMGB1), a protein with increased expression in many autoimmune diseases, and through miR-142-3p, PPARγ thus contributes to maintaining reduced HMGB1 expression [10,83]. Moreover, several studies have demonstrated PPAR-related regulation of histone and DNA modifications in asthma [84] and lupus [85]. PPARs thus regulate immune-related diseases and have the potential to serve as therapeutic targets in these diseases (Table 2).

#### 2.2.1. Asthma

Asthma is an immune disorder characterized by hyper-responsiveness and inflammation of the airways and involving various immune cell types, such as Th2 lymphocytes or eosinophils and inflammatory cytokines. Asthma affects approximately 300 million people worldwide. Although several treatments are available, including corticoids, not all of them are effective and some can have adverse effects in some individuals. Luckily, accumulating evidence is starting to show that PPARs are not only involved in asthma pathogenesis, but could also serve as targets to reduce asthma symptoms [86]. 

A well-known cause of asthma is exposure to nicotine. Human primary lung fibroblasts from smokers and mouse primary lung fibroblasts from mice exposed to nicotine both show reduced PPARγ protein levels [87]. In the nicotine-exposed mice, treatment with the PPARγ pathway activator rosiglitazone restores the expression level of miR-98, a miRNA that negatively regulates the expression of airway remodelling proteins associated with collagen deposition and fibrosis [87]. Similarly, pioglitazone-mediated PPARγ activation in rats inhibits airway smooth muscle cell proliferation and remodelling by supressing the Smad-TGFβ1-miR-21 signalling pathway [88]. Human miR-21 is known to target phosphatase and tensin homolog deleted on chromosome ten (PTEN), thereby promoting airway smooth muscle cell proliferation [88]. However, the proposed beneficial role of PPARs in asthma was brought into question by the recent finding that IgE promotes airway inflammatory remodelling in asthma patients by activating the PPARγ pathway [89]. Moreover, there is currently a lack of specific mouse models for studying the implication of immune cells in asthma, thus impeding the identification of immune regulators linked to PPARs and associated miRNAs such as miR-98.

Research into the PPAR epigenetic regulatory network in asthma has also identified a group of lncRNAs in sputa from patients with eosinophilic asthma (the most common type of asthma) that appear to target and modulate PPAR target-gene mRNAs [90]. However, this report did not specify whether the effect was to increase or decrease PPAR pathway activity, and the samples came from a small pool of just six patients [90]. A study of the leukocyte methylome in asthma patients detected PPARα pathway enriched in differentially methylated regions [84], but the study design did not permit identification of the specific cell types affected.

The proposed anti-inflammatory actions of PPARs in asthma thus point pointing to the therapeutic potential of PPAR agonists in asthma-related disorders [86]. However, although evidence of PPAR-related epigenetic mechanisms in asthma is beginning to emerge, the roles of miRNAs, lncRNAs, and DNA methylation in these processes remains largely unknown.

#### 2.2.2. Systemic Lupus Erythematosus

Systemic lupus erythematosus (SLE) is an autoimmune disease in which dysfunctional immune cells, such as antigen presenting cells, T cells, and B cells, lead to a multiple organ malfunction characteristic of each patient [91]. Among several advances in SLE research, PPARγ has emerged as a promising target, and the PPARγ agonists pioglitazone and rosiglitazone have yielded hopeful results in mouse models of the disease [82,92]. 

Monomethylation of the 20th lysine of histone 4 (H4K20) at the Pparg promoter has been demonstrated to increase the expression of the histone deacetylase HDAC9 [93]. Subsequent analysis of SLE patient samples and mouse models showed that histone modifications at the Pparg promoter influence cytokine and autoantibody production [94]. The authors showed that HDAC9 deletion in mouse CD4+ T cells increased H3K9ac and H3K18ac in the Pparg promoter, prompting a shift in T cell cytokine production towards a more anti-inflammatory class, accompanied by reduced anti-dsDNA autoantibody production by B cells, and therefore protection against proteinuria and renal disease [94]. 

In a very recent study of CD14+ monocytes from SLE patients, Liu Yu et al. reported the emergence of an immunosuppressive M2-phenotype upon TLR-induced epigenetic activation of PPARγ expression [85]. In these experiments, TLR2 activation with the synthetic ligand Pam3CSK4 triggered decreased expression and binding of the deacetylase Sirt1 to the Pparg promoter. ChIP-qPCR revealed that reduced Sirt1 binding leads to increased histone 3 acetylation in the Pparg promoter, with no changes in histone 4 acetylation, resulting in increased PPARγ protein expression and thus allowing the monocytic transition towards a M2 phenotype [85]. These findings are in line with increased Sirt1 expression in the CD4+ T cells of active SLE patients [95]. 

Taken together, these results highlight the importance of the epigenetic modulation of PPARγ in autoimmune diseases such as lupus, the protective role of TLR-Sirt1-PPARγ signalling in SLE, and the therapeutic potential of targeting this pathway and histone deactelyases in SLE. 

#### 2.2.3. Systemic Sclerosis (Scleroderma)

Systemic sclerosis (SSc), or scleroderma, is a rare and severe autoimmune disease featuring diffuse fibrosis and vascular abnormalities in organs, joints, and skin. Of SSc patients, 30% die within 10 years of diagnosis. One of the main challenges of SSc is the rapid worsening of the disease due to uncontrolled inflammation, collagen deposition, and dysregulation of fibroblast growth [96]. 

PPARγ expression is low in SSc lesions [97], and in SSc animal models, ligand activation of PPARγ reduces both TGFβ-dependent fibrogenesis and fibroblast hyperactivation [98]. In line with these findings, PPARγ has been shown to reduce Smad-dependent fibroblast activation and differentiation [99], and PPARγ activation blocks recruitment to DNA of the histone acetyl transferase p300 [100]. p300 is required for interaction with Smad3, activation of the pro-fibrogenic Smad3 pathway [101], and histone 4 hyperacetylation at the Col1a2 locus [100]. PPARγ activation thus leads to Smad3 pathway blockade and reduced collagen production, resulting in diminished inflammation and fibrosis [100,102]. Although no effective therapies have yet been devised for SSc [103], epigenetic-based strategies are being postulated as promising future SSc treatments [104,105,106]. The pharmacological modulation of PPARγ is one of the strategies being considered as a means of epigenetically reducing the fibrotic response in SSc patients.

**Table 2:** PPAR epigenetics in autoimmune diseases.

Condition	PPAR Isoform	Epigenetic Player	Effect	References
Asthma	PPARα	DNA methylation	Human white blood cells showed DNA methylation in several PPAR pathway.	[84]
	PPARγ	miR-21	The profibroti Smad-TGFβ1-miR-21c axis was supress upon PPARγ pioglitazone activation.	[87]
		miR-98	This profibrotic miRNA was downregulated upon PPARγ rosiglitazone activation.	[80]
	Not specified	set of lncRNAs	Modulation of PPAR signalling pathway in sputa from eosinophilic asthma patients.	[90]
Systemic Lupus Erythematosus	PPARγ	H4K20me1 and HDAC9	Decreased H3K9ac and H3K18ac in the Pparg promoter leading to pro-inflammatory T cell cytokines and B cell auto-antibodies.	[93,94]
	PPARγ	Sirt1	Reduced PPARγ expression due to H3 deacetylation, avoiding M2 monocytic transition.	[85]
Systemic sclerosis	PPARγ	p300	Ligand-activated PPARγ blocks histone acetylatransferase p300 avoiding Smad3 pathway activation and Col1a2 locus histone 4 hyperacetylation.	[99,100,101]

### 2.3. Metabolism-Related Diseases

Metabolism-related diseases are a broad class of medical conditions, caused by both genetic and non-genetic defects, which lead to altered metabolic processes. These dysfunctions form a group of diseases that frequently derive from widespread nutritionally poor and unhealthy lifestyles [107]. Overnutrition or low-quality nutrition can lead to a wide range of symptoms converging in the pathologic condition called metabolic syndrome [108]. Some of these symptoms are high blood pressure, high levels of triglycerides, low high-density lipoprotein (HDL) concentrations, increased liver fat, non-alcoholic fatty liver disease (NAFLD), elevated amounts of visceral adipose tissue, insulin resistance and diabetes, high inflammatory state, and even cancer [107,109]. 

Much PPAR research in this area has focused on direct or indirect activation with natural or synthetic ligands [110]. For example, the important role of PPARs in glucose metabolism and effective insulin signaling prompted research into the use of PPARγ-activating TZDs as insulin-sensitizing drugs in T2D [111,112]. More recent approaches have sought to unravel the regulatory networks controlling PPAR expression and function. PPARs clearly play roles spanning many interconnected metabolic disorders. Given the profound effects of transcriptional and epigenetic modulation of PPARs in diverse diseases, new epigenetic targets may have promising therapeutic potential. Here, we focus on the underlying epigenetic mechanisms involving PPARs in three distinct but intimately related metabolic disorders: liver diseases, adipose tissue diseases, and T2D.

#### 2.3.1. Liver Diseases

NAFLD includes a group of liver diseases unrelated to significant alcohol intake. Although the global prevalence and the development of these liver disorders are influenced by ethnicity and geographic origin, there is significant evidence linking NAFLD to poor dietary habits, obesity, adipose tissue dysregulation, and insulin resistance [113]. NAFLD progresses from diet-induced steatosis to a severe inflammatory state, resulting in hepatocyte damage and death that triggers the transdifferentiation of hepatic stellate cells (HSCs) into extracellular matrix-producing myofibroblast-like cells [114]. HSC activation is generally followed by a shift from adipogenesis to a fibrogenic state. This shift is accompanied by a downregulation in the expression of PPARs, which have an anti-inflammatory and protective action in the liver. The shift to fibrogenesis can lead to non-alcoholic steatohepatitis (NASH) and potentially to end-stage liver diseases such as hepatocellular carcinoma.

Several studies have explored epigenetic changes taking place during hepatic metabolic diseases and how they might regulate the expression of PPARs or modulate their binding to promoter and regulatory regions [115,116,117,118] (Table 3). Many epigenetic modifications take place during the progression of steatosis and inflammation and when HSC transdifferentiation begins. For example, many metabolic, proinflammatory, and fibrogenic pathways are regulated by miR-21. This miRNA, which is strongly overexpressed in NASH, represses PPARα expression by direct mRNA targeting and induces HSC activation [119]. Much research into the role PPARs in the hepatic response to dietary fat has focused on the balance between DNA methylation and demethylation and how this determines chromatin accessibility and subsequent changes in gene expression patterns. High dietary fat decreases the methylation of the *Ppara* promoter, resulting in increased PPARα protein expression and the consequent upregulation of carnitine palmitoyl transferase-1 and downregulation of fatty acid synthase, two important lipid metabolism-related enzymes [120,121]. These changes ensure adequate lipid metabolism in response to high dietary fat intake and reveal the important anti-inflammatory role of PPARα in liver diseases and the complex downstream network it controls.

In newborn and suckling mice, PPARα regulates increased liver DNA demethylation and an accompanying increase in the mRNA expression of β-oxidation-related genes [122]. The molecular mechanism underlying this process has not been thoroughly described. Nonetheless, this metabolic transition makes sense given the high dietary fat intake during suckling. A recent study of the livers of fetal and adult offspring of mice fed a high-fat diet during gestation revealed downregulation of the ten-eleven translocation (TET) enzymes TET1 and TET2, together with hypermethylation of *Ppara* and correspondingly lower levels of PPARα protein expression [131]. These findings suggest that dietary alterations during gestation and lactation could downregulate TET enzyme expression in offspring, favouring the hypermethylation of *Ppara* and decreased expression of its lipid metabolism-related target genes. However, further studies are needed to confirm this. TET enzymes require ascorbic acid as a cofactor, and ascorbic acid deficiency during the suckling period increases the hypermethylation of PPARα-dependent lipid metabolism genes such as fibroblast growth factor 21 (*Fgf21*) [132]. FGF21 is a mainly liver-secreted peptide hormone that stimulates adipocytes to take up glucose from the blood [133,134]. In adult mice, fasting-induced FGF21 signalling triggers further epigenetic modifications, such as phosphorylation of the histone demethylase Jumonji-D3 (JMJD3). Phosphorylated JMJD3 interacts directly with PPARα to upregulate the expression of autophagy-related genes [123]. Since this induced process is closely related to triglyceride hydrolysis and ketone body production, PPARα-dependent FGF21–JMJD3 autophagy signalling emerges as an important endocrine regulator and a potential therapeutic target in metabolic disorders [135,136,137].

Other histone modifying enzymes include protein arginine methyltransferase 5 (PRMT5), which regulates gene expression via the dimethylation of histone residues H4R3, H3R8, and H2R3. These methylation marks induce gene silencing through the recruitment of DNA methyltransferase 3a (DNMT3a). PRMT5 is abundant in the liver of fat-fed mice and is implicated in the development of hepatic steatosis [124]. Reduced or annulled PRMT5 expression triggers the overexpression of PPARα and an increased mitochondrial biogenesis [124]. Similarly, the methyltransferase PRMT6 has shown to be a repressor of PPARγ activity [128]. The repression of PPARs by PRMT activity thus presents a further possible target for the treatment of fatty liver.

Although PPARγ is more weakly expressed in the liver than PPARα, it is essential for liver function, and the DNA methylation status of the *Pparg* gene has been identified as a marker of liver disease progression. Analysis of the *Pparg* promoter in plasma cell-free DNA has identified differential DNA methylation patterns in specific CpGs that distinguish between mild and severe fibrosis in NAFLD patients [127]. This cell-free DNA is believed to originate in dying hepatocytes that release their genomic content to the systemic circulation, and thus could provide a noninvasive means of measuring liver status [116]. Taken together, these findings open up new prospective research directions and possibilities for the early diagnosis, screening, and treatment of NAFLD.

PPARγ modulates the expression of lipid uptake and metabolism genes and is a well characterized and important negative regulator of HSC transdifferentiation [125,138]. During this process, downregulation of miR-132 enhances the expression of methyl-CpG binding domain protein 2 (MeCP2), which binds to the 5’ region of *Pparg*, promoting H3K9 methylation and recruitment of the transcriptional repressor heterochromatin protein 1 (HP1α). MeCP2 additionally promotes expression of the H3K27 methyltransferase EZH2 (enhancer zeste homolog 2), generating a repression complex at the 3’ region of *Pparg*. Furthermore, MeCP2 induces the expression of the H3K4 methyltransferase ASH1 (absent small and homeotic disks protein 1), which opposes the action of PPARγ by positively regulating the expression of profibrogenic genes [139,140]. In line with these results, miR-132 was recently linked to human NAFLD [141], and strategies targeting MeCP2 and EZH2 have succeeded in decreasing fibrogenic markers characteristics [142,143]. Additionally, a novel mechanism was shown to promote hepatic lipogenesis through the lncRNA-H19/mi-130a/PPARγ axis [126], becoming a potential target to treat NAFLD.

Other miRNAs involved in PPARγ regulation include miR-29a, which is expressed upon rosiglitazone-induced PPARγ activation in a human HSC cell line and results in the inhibition of fibrosis-related genes [144]. Both miR-29a and miR-652 have been shown to contribute to the resolution of liver fibrosis by modulating the activity of CD4+ T cells and HSCs [145,146]. However, as yet, no relationship has been established between the prevention of HSC activation by miR-652 and PPARγ activity.

PPARγ is also involved in the regulation of adipogenic metabolism by certain demethylases that act as essential modulators of hepatic lipid homeostasis. For example, the H3K9-specific Jumonji demethylases JMJD1A and JMJD2B have been reported to bind to the *Pparg* promoter, and the loss of these enzymes resulted in an increase in the number of H3K9me2 marks in this region, leading to *Pparg* repression and higher levels of fibrosis markers [129]. Conversely, overexpression of these demethylases upregulated *Pparg* expression and increased lipid uptake and intracellular triglyceride accumulation, thus favouring adipogenesis and steatosis [130].

#### 2.3.2. Adipose Tissue Diseases

Evidence accumulated over the past 20 years has established that adipose tissue is an endocrine organ involved in a wide array of metabolic and immune processes [147]. Defects in adipose tissue are typically related to obesity, diabetes and insulin resistance, cardiovascular diseases, cancer, longevity, and even fertility [148,149]. The main transcriptional modulators in adipose tissue are CCAAT/enhancer binding proteins (C/EBP) and PPARγ (specifically PPARγ2), which cooperate in fatty acid uptake and in preadipocyte differentiation to the mature adipocyte phenotype [150,151]. Given the important role of PPARγ in lipid homeostasis, there is intense interest in not only the transcriptional, but also the epigenetic regulation of PPARγ in the development and function of adipose tissue (Table 4 and Table 5).

The methylation status of the *Pparg* promoter undergoes characteristic changes during adipogenesis and obesity. *Pparg* promoter methylation correlates with low expression of PPARγ in preadipocytes of the mouse cell line 3T3-L1 [152], and preadipocyte differentiation to mature adipocytes is accompanied by progressive *Pparg* promoter demethylation as the expression of PPARγ protein increases, whereas obesity is associated with the reverse effect, with *Pparg* methylation increasing as PPARγ expression decreases [152].

**Table 4:** PPARα epigenetics in adipose tissue diseases.

Condition	PPAR Isoform	Epigenetic Player	Effect	References
Adipose tissue diseases	PPARα	Lsd1	Targets PPARα to control beige adipocyte numbers	[153]
		Bta-miR-199a-3p, -154c, -320a and -432	Control lipid metabolism through PPARα	[154]
		miR-519d	Suppresses PPARα protein translation in obese patients	[155]

The expression and function of PPARγ in adipose tissue is determined by insertions of histone variants and histone modifications. A crucial protein in adipocyte differentiation is the complex formed by E1A-binding protein p400 and bromo-containing protein 8 (p400/Brd8). The p400/Brd8 complex can incorporate the histone variant H2A.Z, which preferentially locates within transcriptional regulatory sequences, into the promoter regions of PPARγ target genes [156]. In line with this finding, knockdown of Brd8 or H2A.Z results in cell arrest at the immature preadipocyte stage [156] because the PPARγ target genes involved in differentiation are incorrectly expressed. Histone modifications have been investigated in a genome-wide analysis in mouse and human adipocytes during adipogenesis, demonstrating enrichment of the H3K4me2/me3 and H3K27ac active histone marks in the promoters of *Pparg*1 and 2 [157]. Interestingly, a recent study showed that *Pparg* is repressed by the action of piperine, a major component of black pepper, resulting in the inhibition of various adipogenic genes [158]. In contrast, *Pparg* expression and lipogenesis are enhanced upon H3K4 methylation by the methyltransferases mixed-lineage leukemia proteins 3 and 4 (MLL3 and MLL4), which form a complex with ASC-2 and are recruited by C/EBPβ to the *Pparg* locus [159]. Another study reported that MLL4 induces H3K4me3 marks in the promoters of both C/EBPα and PPARγ through a process requiring the histone methylation regulator PTIP [160]. Moreover, MLL4 itself interacts with some adipogenic TFs, such as tonicity-responsive enhancer binding protein (TonEBP), enabling it to bind the *Pparg* promoter region, increase H3K9me2 marks, and thereby decrease PPARγ expression [161]. Another important methyltransferase in adipocyte differentiation is EZH2, which adds H3K27me marks to the promoter region of the histone deacetylase HDAC9c in adipose tissue, downregulating its expression [162]. Proposals to target EZH2–HDAC9c interaction for the treatment of age-associated osteoporosis and obesity are supported by the report that HDAC9c attenuates adipogenesis by interfering with PPARγ transcriptional activity [163]. Two other methyltransferases of interest are the H3K36 methyltransferase Nsd2 and the lysine methyltransferase 5 (KMT5A, also known as SETD8). Deletion of Nsd2 alters PPARγ target gene expression, adipogenesis, and adipose tissue function [164], whereas KMT5A, a PPARγ target gene expressed during adipocyte differentiation, boosts the expression of PPARγ and the levels of H4K20me marks in other PPARγ target genes in a positive feedback loop [93]. Research has also addressed the role of demethylases in PPARγ regulation in adipose tissue [165], with the histone demethylase JMJD2C reported to downregulate PPARγ transcriptional activation and decrease preadipocyte differentiation, and the H3K9-specific demethylase JHDM2A shown to facilitate the recruitment of PPARγ and RXRα while promoting brown adipogenesis [166,167,168].

Epigenetic analysis of the the *Pparg* gene has revealed increases in H3K9 and H3K27 acetylation marks, paralleling increased PPARγ expression during the differentiation from preadipocytes to mature adipocytes [169]. PPARγ expression is also increased upon the recruitment of C/EBP and the glucocorticoid receptor (GR) to the *Pparg* enhancer by a complex formed between RNA polymerase II transcription subunit 1 (MED1) and the histone acetyltransferase p300 [170]. Another study reported that the *Pparg* promoter and PPARγ target genes are bound by poly(ADP-Ribose)-Polymerase-1 (PARP1), which enhances their expression and thus acts as an adipogenic modulator [171]. However, in contrast with these results, p300 is known to interact with cyclin D1, which inhibits its acetyltransferase activity and thereby reduces *Pparg* expression [172]. These results provide evidence for a central role of PPARγ in the fine epigenetic regulation of adipocyte differentiation, development, and proliferation

Histone deacetylases regulated during adipogenesis include the fasting-induced NAD-dependent histone deacetylase sirtuin-1 (SIRT1). SIRT1 blocks PPARγ activity by docking with the NR co-repressor (NCoR) and the silencing mediator of retinoid and thyroid hormone receptors (SMRT). The resulting complex occupies PPAR binding sites, inhibiting the expression lipogenesis-related genes [173,174]. This finding has prompted interest in SIRT1 as a potential pharmacological target for obesity and obesity-related diseases [173,175]. Recent studies in mouse models of obesity have already demonstrated that HDAC inhibitors stimulate adipose tissue function and oxidative potential, improving the metabolic profile [176,177,178]. Additionally, epigenetic changes upon PPARγ-ligand binding have been studied in relation to their effects on adipogenesis. Rosiglitazone-induced PPARγ activation was found to require the methylcytosine dioxygenase TET2, which is important for demethylation. TET2 enhances the expression of PPARγ target genes and thus participates as an epigenetic regulator and a transcriptional modulator in adipocytes [179]. In 2017, Duteil and colleagues revealed that the lysine-specific demethylase 1 (Lsd1) targeted *Ppara*, maintaining the transcriptional program that sustains beige adipocyte homeostasis. PPARα pharmacological intervention could be used to fight obesity by preventing beige-to-white transition [153].

Research in the past few years has uncovered essential roles of ncRNAs in PPARγ regulation in adipose tissue. The levels of specific ncRNAs have been found to oscillate during adipogenesis and obesity, cell commitment, and adipocyte differentiation. For instance, in vitro studies showed that lncRNA U90926 inhibits *Pparg* promoter activity and therefore decreases its expression [180], whereas nuclear enriched abundant transcript 1 (NEAT1) regulates *Pparg* splicing [181], and the HOX antisense intergenic RNA (HOTAIR) enhances *Pparg* expression and adipocyte differentiation [182]. Another study in obese mice showed that lncRNA taurine upregulated gene1 (TUG1) diminished fatty acid accumulation, insulin intolerance, and inflammation by attenuating miR-204 and promoting GLUT4/PPARγ/AKT pathway [183].

**Table 5:** PPARγ epigenetics in adipose tissue diseases.

Condition	PPAR Isoform	Epigenetic Player	Effect	References
Adipose tissue diseases	PPARγ	U90926	Inhibition of *Pparg* transcription activity	[180]
		NEAT1	Regulation of *Pparg* splicing	[178]
		HOTAIR	Increased expression of PPARγ	[182]
		miR-155, miR-221 and miR-122	Decreased expression of PPARγ in human bone-marrow-derived stromal cells	[184]
		miR-540	Decreased expression of PPARγ in adipose tissue-derived stromal cells	[185]
		miR-27a/b, miR-31, miR-130/b, miR301a, miR-302a and miR-548d5p	Negative regulation of PPARγ and adipogenesis	[186,187]
		miR-103, miR-143, miR-200a, miR-335 and miR-375	Upregulation of *Pparg*	[187,188]
		p400/Brd8 complex	Incorporation of the histone variant H2A.Z, which facilitates the expression of PPARγ target genes	[156]
		MLL3 and MLL4	Complex with ASC-2. Migration to the *Pparg* locus and methylation of H3K4, promoting enhanced *Pparg* expression	[159]
		EZH2	H3K27 methylation in the *Hdac9c* promoter. Enhanced adipogenesis	[162]
		SETD8 (KMT5A)	Enhanced H4K20me marks in PPARγ target genes.	[93]
		JMJD2C	Downregulation of PPARγ transcriptional activation	[166]
		JHDM2A (JMJD1A)	Decreased H3K9me2 marks and facilitated recruitment of PPARγ, RXRα and PGC1α	[167,168]
		Cyclin D1	Interaction with p300 and HDACs to inhibit *Pparg* expression	[172]
		SIRT1	Blocked PPARγ mechanism of action	[173,174]
		LncRNA TUG1 and miR-294	Control fatty acid accumulation through GLUT4/PPARγ/AKT axis	[183]

MiRNAs described to have an epigenetic effect on *Pparg* include miR-155, miR-221, and miR-122. These miRNAs are downregulated during adipogenesis in human bone-marrow-derived stromal cells, and their overexpression results in lower levels of PPARγ [184]. Moreover, bovine fat-enriched miRNAs, Bta-miR-199a-3p, -154c, -320a, and -432, targeted both *Ppara* and *Pparg* in order to control lipid metabolism [154]. Similarly, miR-540 acts as a negative regulator of adipogenesis in adipose tissue-derived stromal cells through binding to the 3′-UTR region of *Pparg* transcripts, blocking their expression [185]. Studies in the 3T3-L1 preadipocyte mouse cell line identified miR-27a/b, miR-31, miR-130/b, miR-301a, miR-302a, and miR-548d5p as negative regulators of *Pparg* expression and thereby inhibitors of adipogenesis [186,187]. In contrast, the expression of miR-103, miR-143, miR-200a, miR-335, and miR-375 accounts for the upregulation of *Pparg* under high-fat diet conditions [187,188,189]. Interestingly, miR-519d has been shown to be upregulated in obese patients and to suppress PPARα protein translation, resulting in an increased lipid accumulation during pre-adipocyte differentiation [155].

Together, these results demonstrate the importance and complexity of the epigenetic regulation of PPARs in the control of adipogenesis and adipocyte differentiation in homeostatic and pathological conditions. Since the mechanisms by which adipocytes acquire their specific identity are well known, the quest for new therapeutic applications appears to be very promising. Although some of studies cited here were carried out in human preadipocytes and human multipotent adipose-derived stem cells, most research has been performed in adipocytes from mouse models of obesity. Further research into the epigenetic control of PPARs in human studies is thus needed to move the field towards therapeutic applications in obesity and adipose tissue disorders.

#### 2.3.3. Insulin Sensitivity and Resistance: Type 2 Diabetes

Diabetes is a metabolic disorder characterized by an inability to properly clear glucose from the blood. The most common form is T2D, in which two related features converge: insufficient insulin production by pancreatic β-cells and progressive insulin resistance [190]. T2D is intimately associated with obesity, inflammation, ageing, and steroid use, and over the past decades its incidence has worryingly increased in children [191,192,193]. Although research has traditionally focused on insulin signaling defects, some studies have emphasized the transcriptional and epigenetic basis of chronic inflammation in insulin resistance and T2D [194] (Table 6), and others have identified NRs, such as the glucocorticoid and vitamin D receptors, as common mediators of insulin resistance [195].

Although NRs require activating ligands, some researchers have concluded that post-translational modifications such as acetylation increase NR activity in the absence of external ligand [196]. Some histone deacetylases have been implicated in post-translational modifications of PPARs and their activity. High expression of the deacetylase HDAC3 correlated with high levels of proinflammatory markers and insulin resistance in peripheral blood mononuclear cells from T2D patients and hepatocytes from fat-fed E3 rats, which develop metabolic syndrome [197,198]. Inhibition of HDAC3 in adipocytes increased PPARγ acetylation and the expression of PPARγ target genes, including adipokines and adipocyte protein 2, resulting in decreased insulin resistance. These adipokines include adiponectin, which facilitates hepatic glucose output, and leptins, which are important regulators of feeding behaviour [196,199]. Adipose tissue-specific knockout of SIRT 1 triggers a hyperacetylated PPARγ state and enhanced PPARγ activity, leading to increased insulin sensitivity [175]. These results suggest that HDAC inhibitors have the potential to improve insulin sensitivity through a variety of actions. For example, HDAC inhibitors might release PPAR binding sites, as described for SIRT1, and promote maintenance of the acetylated state of PPARs and PPAR target genes. These inhibitors could also stimulate significant PPARγ activation. Recent studies have begun to explore the therapeutic potential of HDAC inhibitors in insulin resistance and obesity [200,201,202]. However, their application to human disease requires further research.

T2D is also closely related to immunity. During diabetes, adipose tissue macrophages (ATMs) are activated and shift to a pro-inflammatory phenotype, contributing to the propagation of the altered metabolic state by expressing the pro-inflammatory cytokines TNFα, IL-6, and MCP-1 [203,204]. Macrophage activation during T2D is in part mediated by epigenetic mechanisms [205]. The regulation of ATM alternative activation and insulin sensitivity correlate with PPARγ activation [206,207,208], and ATM alternative activation is held in check by DNA methylation at the *Pparg* promoter. DNA methylation blockade at the *Pparg* promoter boosts macrophage alternative activation, whereas DNA hypermethylation promotes inflammatory responses and insulin resistance [209]. In another study, DNMT3b downregulation in ATMs was found to promote an anti-inflammatory state and enhanced insulin sensitivity, revealing the contribution of DNMT3b-mediated methylation at the *Pparg* promoter to increased inflammatory conditions and insulin resistance [210]. Studies have also reported the contribution of other DNMTs to the epigenetic control of PPARγ target genes. For instance, hypermethylation of *FGF21* by DNMT3a in human adipocytes decreased its expression and correlated with insulin resistance in patients [211]. In another study, methylation of the adiponectin promoter by DNMT1 reduced adiponectin expression in obese mice, and DNMT1 inhibition increased insulin sensitivity and ameliorated glucose intolerance [212]. DNMT inhibitors are thus able to lower DNA methylation that directly affects *Pparg* and PPARγ target genes, identifying these inhibitors as a promising potential treatment for T2D.

The adipogenesis inhibiting miRNA miR-27a has also been reported to promote insulin resistance [213], acting as a glucose metabolism mediator that regulates the PI3K–Akt–GLUT4 signalling pathway by targeting the 3’UTR region of *Pparg* transcripts, promoting insulin resistance [213]. MiR27a is also upregulated during obesity and induces ATM proinflammatory activation by targeting *Pparg* [214].

**Table 6:** PPAR epigenetics in type 2 diabetes.

Condition	PPAR Isoform	Epigenetic Player	Effect	References
Insulin sensitivity and resistance: Type 2 Diabetes	PPARγ	miR27-a	Target of *Pparg* transcripts, promoting insulin resistance. Induction of inflammatory ATM activation in obesity	[213,214]
		HDAC3	Decreased expression of PPARγ in E3 rat livers. Correlated with inflammation and insulin resistance	[196,197,198]
		SIRT1	Control of the PPARγ acetylation status and its activity	[175]
		DNMT3b	*Pparg* promoter methylation. Increased inflammatory macrophage activation and insulin resistance	[209,210]
		DNMT3a	Fgf21 hypermethylation in human adipocytes, insulin resistance	[211]
		DNMT1	Adiponectin promoter methylation in obese mice. Glucose intolerance	[212]

Further epigenetic studies have focused on the PPAR coactivator 1α (PGC1α). This protein binds and modulates the activity of PPARγ and PPARα, thereby indirectly regulating the expression of PPAR target genes and functions [215,216]. Like PPARγ, PGC1α can be regulated by reversible acetylation. Its protein sequence contains 13 lysine acetylation sites, and acetylation/deacetylation of these sites depends on the cell energy state [217]. PGC1α can be activated by deacetylation mediated by SIRT1 [218,219]. This activation promotes the expression of PPAR target genes and increased expression of gluconeogenic genes [220]. In contrast, PGC1α is inactivated by acetylation by p300, SRC1/3, GCN5, or hepatic PCAF, producing the opposite effect [220]. Epigenetic changes thus not only control *Pparg* expression directly, but also regulate the availability and activity of obligate PPARγ coactivators. These studies increase the relevance of *Pparg* epigenetic modulation and underline the importance of continuing to develop new therapeutic approaches to apply these observations to the treatment of T2D.

## 3. Conclusions

Despite the importance of PPARs in the control of inflammation and lipid homeostasis in different disease contexts, efforts to decipher the diversity of PPAR-related epigenetic modulation are still at an early stage. This review provides a broad overview of PPAR biology and epigenetics in different diseases. PPARs have a complex and tightly regulated transcriptional network that when dysregulated can lead to disease conditions such as metabolic disorders, autoimmune diseases, or cancer. Although research in this area has characterized several factors of the PPAR regulatory network, the epigenetic effectors and regulators remain largely unknown. For instance, many studies discussed have established a correlation between PPAR and epigenetics in different diseases but have failed to establish a clear causal relationship. Nonetheless, the current evidence establishes that cancer-related, immune, and metabolic disorders have an epigenetic regulatory basis, in which PPARs act as central regulators of inflammation, fibrosis, immune responses, as well as lipid and glucose homeostasis. Some lines of research suggest a potential for therapeutic strategies based on PPAR epigenetics. For instance, HDAC and DNMT inhibitors could serve as therapies in PPAR-dependent inflammatory diseases such as obesity or cancer. Moreover, some PPAR network epigenetic effectors such as miRNAs could be used as early biomarkers of specific disorders. The PPAR epigenetic network is a fascinating emerging field of study that is beginning to identify promising targets for the treatment of cancer, immune, and metabolic disorders.

## Figures and Tables

**Table 3:** PPAR epigenetics in non-alcoholic steatohepatitis.

Condition	PPAR Isoform	Epigenetic Player	Effect	References
NASH	PPARα	miR-21	Diminished PPARα expression and activation of HSCs in obesogenic models	[119]
		TET1 and TET2	Downregulated enzymes under high fat diet conditions, promoting *Ppara* hypermethylation	[121]
		Ascorbic acid	Cofactor of TET enzymes. Its lack promotes PPARα target genes hypermethylation	[122]
		JMJD3	Phosphorylated upon fasting-induced FGF21 signaling. Direct interaction with PPARα for the upregulation of autophagy-related genes	[123]
		PRMT5	Downregulation of *Ppara* expression	[124]
	PPARγ	miR-132	miR-132 downregulation induces the expression of MeCP2 in HSCs	[125]
		miR-29a	Expressed upon Rosiglitazone-mediated PPARγ activation. Repression of profibrotic genes	[126]
		MeCP2	H3K9 and H3K27 methylation and HP1α repressor recruitment in *Pparg* locus of HSCs. MeCP2 also induces the expression of EZH2 and ASH1 in HSCs.	[125]
		*Pparg* promoter CpG methylation	Downregulation of PPARγ. Potential non-invasive fibrosis marker in cell-free DNA in plasma.	[127]
		PRMT6	Repression of PPARγ activity	[128]
		JMJD1A and JMJD2B	Upregulation of *Pparg* and increased lipid uptake	[129,130]
		LncRNA-H19	Control of hepatic lipogenesis through mi-130A/PPARγ axis	[126]
